# Crimean-Congo Hemorrhagic Fever Virus Genome in Tick from Migratory Bird, Italy

**DOI:** 10.3201/eid2507.181345

**Published:** 2019-07

**Authors:** Elisa Mancuso, Luciano Toma, Andrea Polci, Silvio G. d’Alessio, Marco Di Luca, Massimiliano Orsini, Marco Di Domenico, Maurilia Marcacci, Giuseppe Mancini, Fernando Spina, Maria Goffredo, Federica Monaco

**Affiliations:** Istituto Zooprofilattico Sperimentale dell’Abruzzo e del Molise “G. Caporale,” Teramo, Italy (E. Mancuso, A. Polci, S.G. d’Alessio, M. Orsini, M. Di Domenico, M. Marcacci, G. Mancini, M. Goffredo, F. Monaco);; Istituto Superiore di Sanità, Rome, Italy (L. Toma, M. Di Luca);; Istituto Superiore per la Protezione e la Ricerca Ambientale, Bologna, Italy (F. Spina)

**Keywords:** Crimean-Congo hemorrhagic fever virus, CCHFV, Hyalomma rufipes, Saxicola rubetra, Ventotene, Italy, tickborne diseases, vector-borne infections, viruses, ticks, Crimean-Congo hemorrhagic fever

## Abstract

We detected Crimean-Congo hemorrhagic fever virus in a *Hyalomma rufipes* nymph collected from a whinchat (*Saxicola rubetra)* on the island of Ventotene in April 2017. Partial genome sequences suggest the virus originated in Africa. Detection of the genome of this virus in Italy confirms its potential dispersion through migratory birds.

Crimean-Congo hemorrhagic fever virus (CCHFV) is a vectorborne virus responsible for severe illness in humans, whereas other mammals usually act as asymptomatic reservoirs. The virus is transmitted through tick bites or by direct contact with blood or body fluids of infected vertebrate hosts. CCHFV, an *Orthonairovirus* within the Nairoviridae family, has a negative-sense tripartite RNA genome characterized by high genetic diversity. The sequences of the circulating strains cluster in 6 genotypes (I–VI) reflecting their geographic origin; worldwide distribution is the result of efficient dispersion through migratory birds, human travelers, and the trade and movement of livestock and wildlife (*1*,*2*). In Europe, CCHFV distribution was limited to the Balkan region until 2010, when the virus was identified in ticks collected from a red deer (*Cervus elaphus*) and, 6 years later, in 2 autochthonous human cases in the same region of Spain (*3*). Sequences from the Iberia strains clustered in the Africa genotype III (*4*), supporting the hypothesis of CCHFV dispersion through ticks hosted by migrating birds.

The role of birds in the potential spread of the virus was confirmed by CCHFV detection in ticks collected from migratory birds in Greece in 2009 (*5*) and Morocco in 2011 (*6*). Because Italy hosts an intense passage of birds migrating along major routes connecting winter quarters in Africa and breeding areas in Europe, the country is potentially exposed to the risk for virus introduction. We report the detection of CCHFV RNA in a tick collected in Italy from a migratory bird.

We conducted tick sampling during March–May 2017 on the island of Ventotene, where a ringing station has been operating since 1988 as part of the Small Islands Project, a large-scale and long-term effort to monitor spring migrations of birds across the central and western Mediterranean. We ringed 5,095 birds and checked ≈80% for ectoparasites. We collected 14 adults, 330 nymphs, and 276 larvae from 268 passerines belonging to 28 species; 18 species were trans-Saharan migrants. We stored ticks in 70% ethanol until morphologic identification and assignment to a genus or, whenever possible, a species (*7*). We then individually homogenized the ticks and extracted nucleic acids by using the Maxwell16LEV simplyRNA Blood Kit with a Maxwell16 Instrument (Promega, https://twitter.com/promega). We assayed purified nucleic acids for the presence of CCHFV RNA and to refine the morphologic identification of the immature ticks. We used the RealStar CCHFV RT-PCR Kit 1.0 (Altona Diagnostics, https://www.altona-diagnostics.com) to reveal viral RNA and conducted molecular tick species identification by sequencing 340 bp of the 12S rDNA sequence (*8*). We detected CCHFV RNA in a half-fed nymph of *Hyalomma rufipes*, a member of the *H. marginatum* complex, collected from a whinchat (*Saxicola rubetra*), a trans-Saharan migratory bird distributed across Europe and western Asia that winters mainly in central Africa.

We further sequenced the sample by using the Illumina Next 500 platform (Illumina, https://www.illumina.com) with a slightly modified sequence-independent single-primer amplification protocol. We cleaned and denovo assembled raw reads by using SPADES 3.11 (http://cab.spbu.ru/software/spades). We identified 18 partial but reliably assembled contigs (97%–99% identity) that could be definitely assigned to CCHFV. We aligned partial sequences from small (485 bp) and medium (1,374 bp) segments with 13 virus sequences representative of the 6 circulating virus genotypes. We performed cladistics analysis by using MEGA7 (https://www.megasoftware.net). Whatever the genome segment involved, the cladograms revealed that the strain identified in Italy originated in Africa and had a close affinity with those strains segregated in genotype III (Figure) but excluded a direct introduction from eastern Europe.

**Figure:**
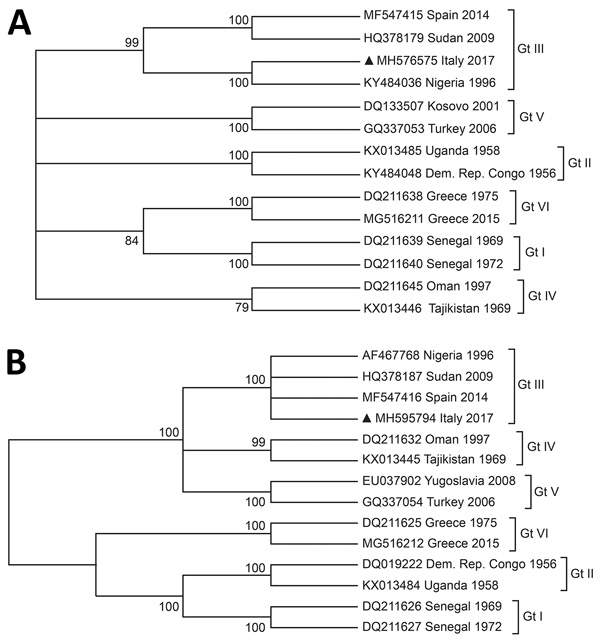
Cladistic relationship of Crimean-Congo hemorrhagic fever virus gene found in a *Hyalomma rufipes* nymph collected from a whinchat (*Saxicola rubetra)* in Italy (black triangles) with strains representative of the circulating genotypes. The analysis is based on partial sequences of the small segment (A) and medium segment (B) of the virus genomes. We used the neighbor-joining method based on the Tamura-Nei model. Percentage of replicate trees in which the associated taxa clustered together in the bootstrap test (1,000 replicates) is shown next to the branches. The analysis involved 14 nucleotide sequences. Codon positions included were first, second, third, and noncoding. All positions containing gaps and missing data have been eliminated. A total of 485 positions (small segment) and 1,374 positions (medium segment) were included in the final dataset. GenBank accession numbers are provided. Gt, genotype.

The origin of the introduction of this virus is further supported by the geographic distribution of the infected tick, identified as *H. rufipes*. Adults of this species have been sporadically collected in Europe (*9*,*10*) but are mainly distributed in sub-Saharan Africa, whereas *H. marginatum* ticks are autochthonous in many Mediterranean countries. *H. marginatum* and *H. rufipes* ticks are competent vectors for CCHFV (*2*) and are characterized by a 2-host cycle, with birds usually acting as hosts of immature stages. Larvae feed on birds and molt to become nymphs, remaining on the same avian host up to 26 days, a period that usually lasts until the trans-Saharan migrating birds have reached Europe (*1*). Thus, the half-fed nymph probably was attached to the whinchat when migration started. Nymphs drop off the bird only after completion of the blood meal and molt on the ground before attaching to their second, final hosts, which are usually large mammals, including humans.

Although detection of virus genome does not necessarily imply the presence of live virus that is able to spread locally, our findings, consistent with the recent autochthonous cases in Spain, underscore the need to monitor any introduction and circulation of CCHFV in southwestern Europe. Such monitoring should focus on sites where migrants rest or nest and where a local population of competent ticks and their hosts interact. Raising awareness of possible outbreaks should also include specific surveillance and contingency plans focused on categories of persons and animals at elevated risk for CCHFV infection.
